# Posterior osteosynthesis with a new self-designed lateral mass screw-plate system for unstable atlas burst fractures

**DOI:** 10.1186/s12891-023-06209-z

**Published:** 2023-02-09

**Authors:** Kun Yang, He-gang Niu, Hui Tao, Chang Liu, Yun Cao, Wei Li, Jing-jing Zhang, Cai-liang Shen, Yin-shun Zhang

**Affiliations:** Department of Orthopedics, the First Affiliated Hospital of Anhui Medical University, No.218 Jixi Road, Hefei, 230022 Anhui Province China

**Keywords:** Atlas fracture, C1 fracture, Open reduction and internal fixation (ORIF), Osteosynthesis, Transverse atlantal ligament (TAL)

## Abstract

**Background:**

In the treatment of unstable atlas fractures using the combined anterior–posterior approach or the posterior monoaxial screw-rod system, factors such as severe trauma or complex surgical procedures still need to be improved despite the favourable reduction effect. This research described and evaluated a new technique for the treatment of unstable atlas fracture using a self-designed lateral mass screw-plate system.

**Methods:**

A total of 10 patients with unstable atlas fractures using this new screw-plate system from January 2019 to December 2021 were retrospectively reviewed. All patients underwent posterior open reduction and internal fixation (ORIF) with a self-designed screw-plate system. The medical records and radiographs before and after surgery were noted. Preoperative and postoperative CT scans were used to determine the type of fracture and evaluate the reduction of fracture.

**Results:**

All 10 patients were successfully operated with this new system, with an average follow-up of 16.7 ± 9.6 months. A total of 10 plates were placed, and all 20 screws were inserted into the atlas lateral masses. The mean operating time was 108.7 ± 20.1 min and the average estimated blood loss was 98.0 ± 41.3 ml. The lateral mass displacement (LMD) averaged 7.1 ± 1.9 mm before surgery and almost achieved satisfactory reduction after surgery. All the fractures achieved bony healing without reduction loss or implant failure. No complications (vertebral artery injury, neurologic deficit, or wound infection) occurred in these 10 patients. At the final follow-up, the anterior atlantodens interval (AADI) was 2.3 ± 0.8 mm and the visual analog scale (VAS) was 0.6 ± 0.7 on average. All patients preserved almost full range of motion of the upper cervical spine and achieved a good clinical outcome at the last follow-up.

**Conclusions:**

Posterior osteosynthesis with this new screw-plate system can provide a new therapeutic strategy for unstable atlas fractures with simple and almost satisfactory reduction.

## Background

Located at the craniocervical junction, the atlas consists of two wedge-shaped lateral masses connecting the anterior and posterior arches, whose connection is prone to fracture and lateral displacement under axial pressure due to anatomical and biomechanical conditions. Previously, atlas fractures could be divided into stable and unstable fractures according to the integrity of the transverse ligament of atlas [1, 2]. Atlas fractures are considered to be stable isolated unilateral or bilateral posterior arch fractures or unilateral anterior arch fractures without TAL injury [3, 4]. Stable atlas fractures are generally treated conservatively, while unstable atlas fractures can be treated nonsurgically or surgically. Non-surgical treatment might be ineffective and may lead to nonunion or malunion of the fracture. On this basis, surgical treatment is considered as the main clinical choice [5–7]. Atlantoaxial fusion or occipitocervical fusion is a traditional surgical method for the treatment of unstable atlas fractures. However, patients' quality of life may be seriously affected by the postoperative loss of upper cervical spine motor function and accelerated degeneration of adjacent segments [8].

Scholars worldwide have proposed an ideal surgical method for the unstable C1 fractures, including anterior transoral approach [9–11] and posterior open reduction and internal fixation (ORIF) [12–16], which can not only stabilize the fracture, but also preserve the function of C0-C1-C2 joint. Despite the satisfactory reduction, the anterior transoral surgery is difficult to be widely promoted due to unfamiliar approach and high infection rate. Hence, more surgeons prefer the posterior approach. Nevertheless, previous studies have shown that satisfactory anatomical reduction of the anterior arch could not be easily achieved using a screw-rod or a screw-plate system. Zhang et al. [17] and Rainer G. Ale et al. [18] firstly proposed and systematically described the posterior osteosynthesis with a monoaxial lateral mass screw-rod system in the treatment of atlas fracture, which could achieve a satisfactory anatomical reduction of anterior arch atlas fractures. However, the operation was complicated and some patients developed postoperative symptoms of excessive occipital nerve stimulation due to the large nail cap.

In the present study, we designed a posterior low-profile screw-plate system for the treatment of atlas fractures, and retrospectively analyzed the outcomes of 10 patients. The result showed that, this technique simplified the operation, improved surgical safety and reduced surgical trauma, leading to almost satisfactory reduction of anterior and posterior archs and lateral mass of the atlas.

## Methods

### Clinical data

This study was approved by the hospital Ethics Committee. Each patient signed an informed consent before sugery. A total of 10 patients with unstable atlas fracture underwent posterior osteosynthesis with a lateral mass screw-plate system in our hospital from January 2019 to December 2021 were retrospectively analyzed. Each of them presented neck pain, stiffness, and limited neck range of motion, without neurologic deficit. Patients with axial or occipital condyle adjacent level fractures, chronic atlas fractures and nonunion of atlas fractures were excluded. All patients underwent preoperative anteroposterior open-mouth and lateral radiographs, computed tomography (CT) scan and three-dimensional reconstructions of the upper cervical spine. Magnetic resonance imaging (MRI) before the operation was examined to evaluate the integrity of the ligament elements at the  C0-C1-C2 junction, especially the injury of the TAL. The lateral mass displacement (LMD) of the atlas was calculated from the coronal reconstructed view of CT scan.

### Surgical technique

**Fig. 1:**
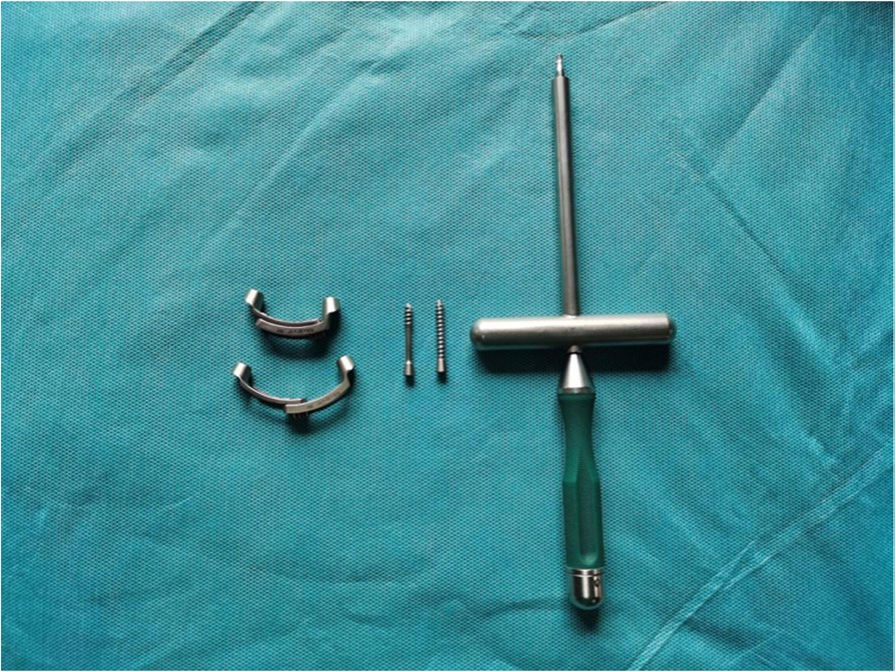
The system is composed of two arc-shaped fixed plates connected by adjusting nuts, full thread or half thread screws and the locking device

**Fig. 2:**
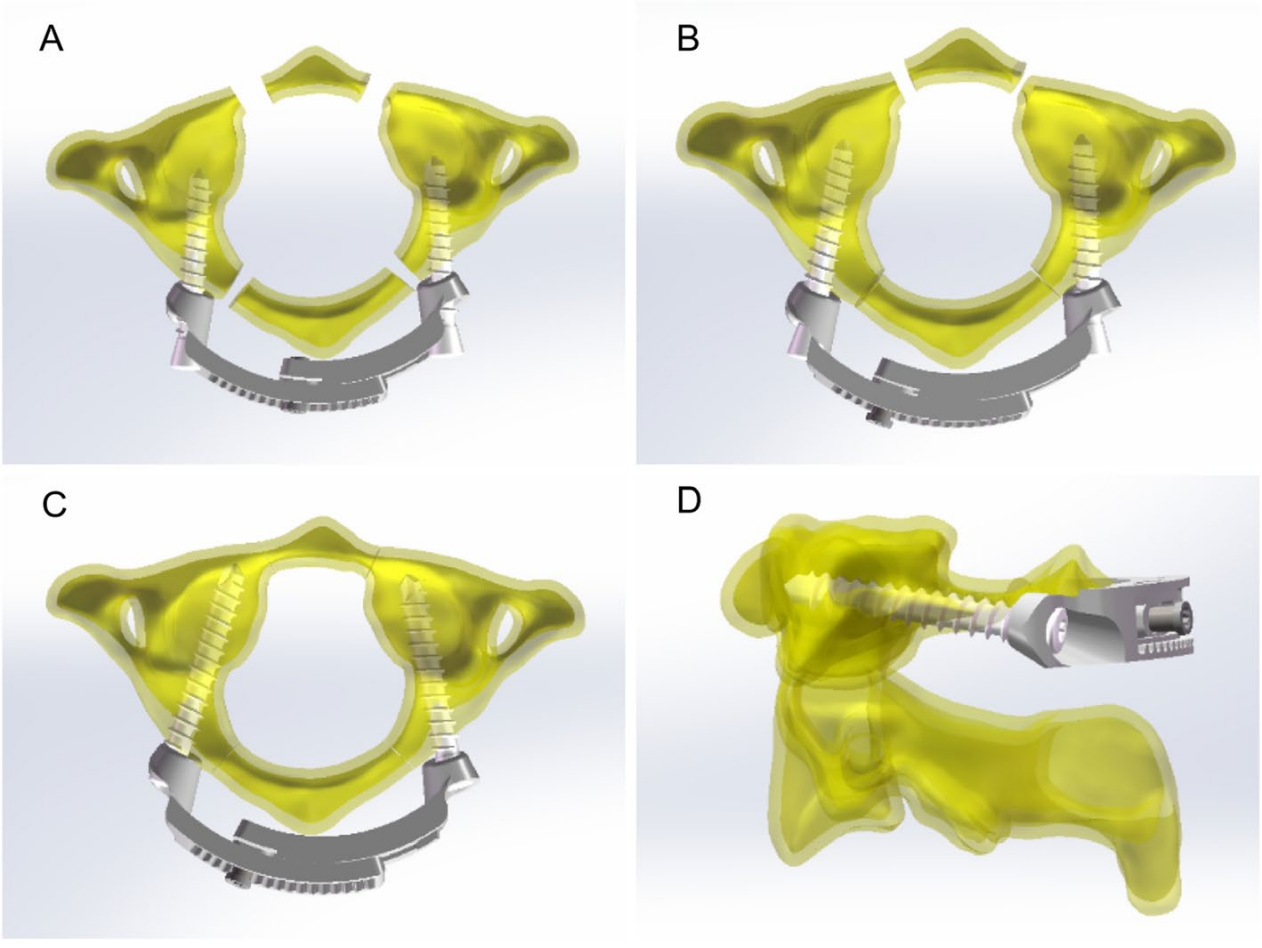
Schematic diagram of fracture reduction process. **A** The pedicle screws were implanted through the lateral screw holes of the arc-shaped plates on both sides, and remained unlocked temporarily. The axial inclination angle of the oblique conical screw hole (15°-20°) was slightly larger than the actual angle of the screw path. **B** By rotating the adjusting nut with a special instrument, the two arc-shaped plates slided each other, driving the atlas lateral mass to move inward, locking the adjusting nut, and realizing reduction of posterior atlas arch fracture through compression under direct vision. **C** Then the bilateral pedicle screws were gradually tightened. When locking the screws, the anterior part of the screw drove the lateral mass of the atlas to rotate forward and inward, so as to achieve compression reduction of the anterior atlas arch fracture. **D** Side view of this screw-plant system. The axis direction of the screw hole and the central plane direction of the plate had a certain angle (10°-15°), so that the plate could be directly attached to the posterior arch of atlas after the screw placement, avoiding the upper edge of the plate from squeezing the posterior arch of atlas, and also reducing the compression of the plate on the axial lamina

Each patients was placed in a prone reverse-Trendelenburg position via a Mayfield head holder with skull traction, which contributed to not only partial reduction, but also favourable posterior arch exposure. Antibiotics were routinely administered 30 minutes preoperatively to prevent infection. A standard posterior midline incision was made from the occiput to the C3 spinous process to expose the C1 subperiosteal posterior arch about 30 mm lateral from the midline, and the extensor muscle insertion on the C2 spinous process was preserved as much as possible. A high-speed power drill was applied to make a screw path before inserting the screws into the posterior arch of the C1 using the notching technique [19]. The ideal entry points, screw path, and screw length were planned according to the preoperative 3D-CT measurements. The pedicle screws were implanted through the lateral screw holes of the arc-shaped plates on both sides, and remained unlocked temporarily. By rotating the adjusting nut with a special instrument (Fig. 1), the two arc-shaped plates slided towards each other to drive the atlas lateral mass to move inward. By locking the adjusting nut, the compression reduction of the posterior atlas arch fracture under direct vision was achieved. Then the bilateral pedicle screws were gradually tightened. Due to the conical structure of the screw tail, the relative position would gradually match the conical hole on the arc-shaped plate. When locking the screws, the anterior screw drove the lateral mass of the atlas to rotate forward and inward, so as to achieve compression reduction of the anterior atlas arch fractures (Fig. 2 A-C). Half-thread screws were applied as lag screws for reduction of the lateral mass coronal split fractures. This product was designed by our team and produced by Fule Company in Beijing, China. Intraoperative fluoroscopic posteroanterior view or C-arm 3D imaging confirmed that fracture reduction was almost satisfactory.

### Postoperative treatment

All patients were encouraged to mobilize on the first postoperative day. Antibiotics were routinely administered for 48 h after surgery. Postoperative CT scanning was performed within 1 week to evaluate the efficiency of fracture reduction and the position of C1 screw-plate. External immobilization was performed with a Philadelphia cervical collar for 6 weeks after the operation. After 6 months, the upper cervical spine was scanned by CT and 3D reconstructions to evaluate the fusion. In addition, the anterior atlantodens interval (AADI) was evaluated on the flexion view of the lateral flexion–extension radiographs and a 10-point visual analog scale (VAS) score was used to evaluate the level of neck pain at the final follow-up.

## Results

**Fig. 3:**
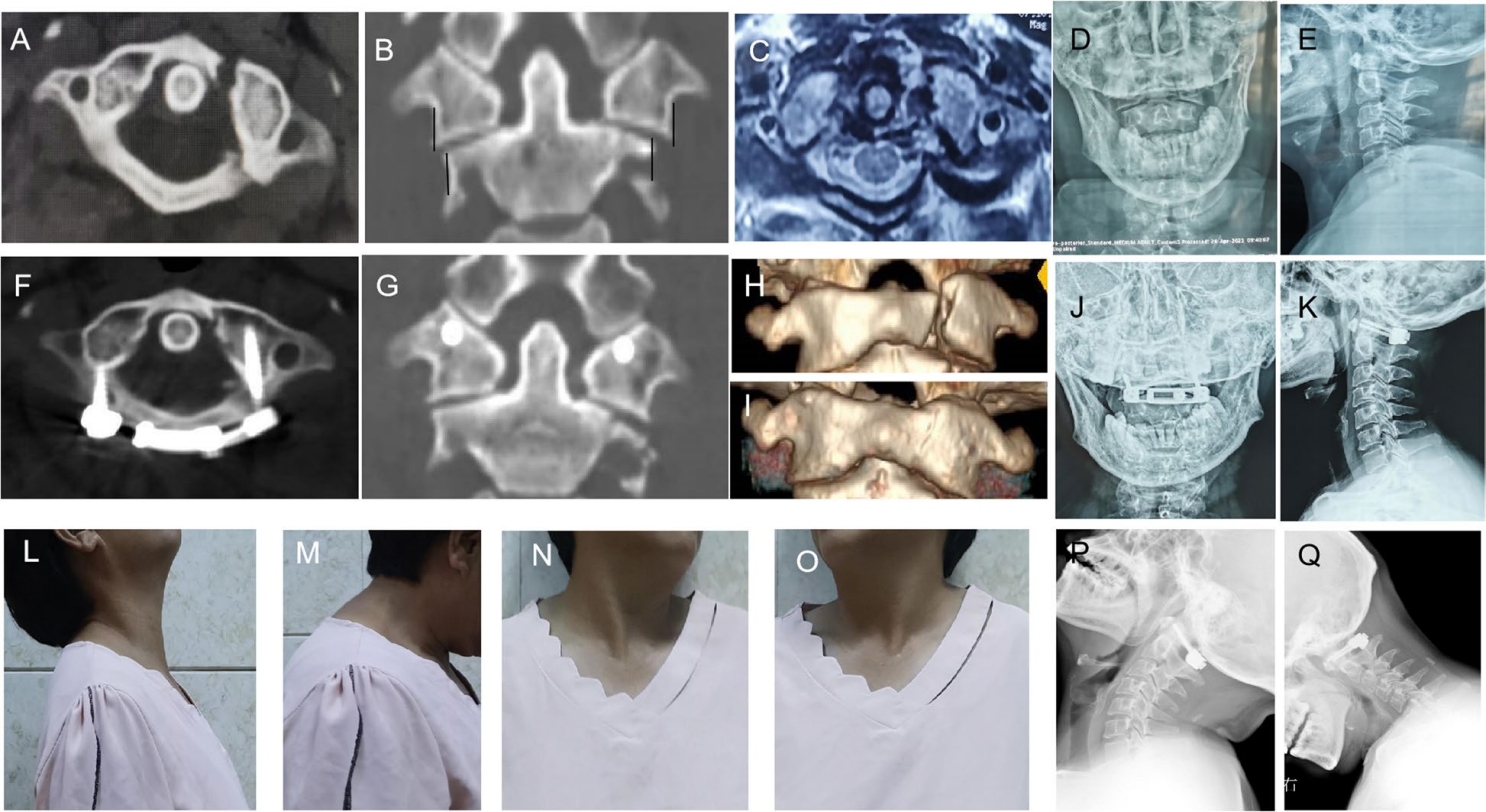
A 50-year-old woman with C1 left anterior and posterior arch fractures (**A**, **H**), lateral mass displacement (**B**, **D**, **E**) and Type II TAL injury (**C**). Postoperative CT scans and 3-D reconstructed images revealed satisfactory reduction (**F**, **G**, **I**).The lateral and open-mouth X-ray image (**J**, **K**) at 2 months after surgery revealed that the screws of the atlas lateral masses were placed sufficiently, and the fixation system was not loose. The patient completely recovered the full range of motion of the cervical spine without pain at the last follow-up (**L-O**). The lateral radiograph of the fexion-extension cervical spine demonstrated good atlantoaxial stability without dislocation (**P-Q**)

Base on Landells and Van Peteghem classifification system [20], there were 7 type II and 3 type III fractures in all 10 patients. Transverse atlantal ligament (TAL) injury was found in 8 of the 10 patients: one of type I (a disruption of the midportion of the transverse ligament) and seven of type II (fractures and avulsions involving the tubercle for insertion of TAL on the C1 lateral mass) based on Dickman’s classification. The remaining two patients about the TAL injury diagnosis were uncertain. All patients were followed up from 8 to 42 months, with an average of 16.7 ± 9.6 months. The preoperative LMD averaged 7.1 ± 1.9 mm and was restored completely after surgery. A total of 10 plates were placed, and all 20 screws were inserted into the atlas lateral masses. The mean operating time was 108.7 ± 20.1 min, and the average estimated blood loss was 98.0 ± 41.3 mL. Computed tomography confirmed that fusion was achieved in all cases 6 months after surgery. No screws or plates were loose or broken in all patients. The AADI was 2.3 ± 0.8 mm and the VAS was 0.6 ± 0.7 on average at the last follow-up. All patients preserved almost full range of motion of the upper cervical spine. No vascular or neurologic complication was noted, and all patients had a good clinical outcome (Fig. 3, Table 1).

**Table 1:** Clinical data of C1 burst fracture patients treated with posterior osteosynthesis with a new self-designed lateral mass screw-plate system

Variables	Sex (Male,n)	Age (years)	Follow-up time (months)	Type of fractures (n)		TAL injury (n)			Surgical time (min)	Blood loss (ml)	Preo-LMD (mm)	AADI (mm)	VAS
				II	III	I	II	Uncertain					
	10(9)	47.0 ± 9.7	16.7 ± 9.6	7	3	1	7	2	108.7 ± 20.1	98.0 ± 41.3	7.1 ± 1.9	2.3 ± 0.8	0.6 ± 0.7

*Preo-LMD* Preoperative lateral mass displacement, *AADI* Anterior atlantodens interval, *VAS* Visual analog scale

## Discussion

Atlas fracture was first proposed by Cooper in 1822, and later summarized and generalized by Jefferson [2]. The incidence of atlas fracture accounts for 1–2% in spinal fractures, and 2–13% in cervical fractures [2], which are mostly caused by traffic accidents or high falls. Due to its low incidence, atlas fracture occupies a small proportion in the literature, with varied treatment.

The key treatment of atlas fracture lies in whether the fracture is stable. Traditionally, the stability of atlas fracture is determined by the integrity of TAL. Stable atlas fractures with intact transverse ligaments can be treated conservatively, such as rigid cervical fixation, which can achieve satisfactory outcomes [21]. It remains controversial in the treatment of unstable atlas fractures with incomplete transverse ligaments. In the early stage, rigid collars, suboccipital mandibular immobilizer braces, and halo ring-vest orthoses are commonly used for 8 to 12 weeks. However, non-surgical treatment may result in poor reduction of atlas fracture, as well as non-union and malunion of fracture, leading to cranial demystification and neurologic sequels [21], which may be unbearable for patients and they will eventually choose surgical treatment. Dvorak et al. [22] published the first study in 2005, attempting to address the quality-of-life issues in patients with isolated atlas fractures. The majority of patients have difficulty returning to their pre-injury level of function, and the prognosis of unstable atlas fractures was worse than that of stable ones, suggesting that non-surgical treatment may not be an ideal surgical option. Therefore, surgery is considered as the main treatment for unstable atlas fractures. Due to the overemphasis on the instability caused by TAL injury, C1-2 or even C0-2 fusion was selected for traditional surgical treatment, which sacrificed C1-2 rotation and even C0-1 flexion and extension function, and significantly reduced patients' postoperative quality of life.

Most of the literatures [1, 2] reflect a consensus that the transverse ligament is the primary stabilizing component concerning stability assessment. Notably, the ligaments between C0-C1-C2 bony structures are also vital to the stability of this region. In 2011, Li et al. [12] put forward the “Buoy phenomenon” hypothesis in the treatment of unstable atlas fractures with TAL injury using a screw-rod system. Li et al. thought it paramount to restore the C0–C2 height to recreate the ligamentous tension band, which could restore the normal relationship of C0-C1-C2 ligament complex, tighten the longitudinal ligaments, and maintain atlantoaxial vertebral stability when appropriate bilateral transverse compression force was gently applied for further reduction. Biomechanical studies [23–25] showed that, in the atlas unstable fracture model, there was no significant loss of atlantoaxial three-dimensional stability after C1-ring osteosynthesis with TAL rupture, which was feasible for surgery. Therefore, TAL incompetence may not be a contraindication of ORIF in C1 unstable burst fractures [16].

In order to preserve more C0-1 and C1-2 functions, Ruf et al. [9] reported 6 cases of unstable atlas fractures treated by transoral approach. All cases achieved postoperative bone healing and preserved C1-2 rotation and C1-2 flexion and extension functions. No instability was found in the postoperative review.

Zou et al. [10] and Tu et al. [11] also obtained similar results for anterior screw-plate reduction of atlas fractures. Despite the satisfactory reduction, the anterior transoral approach is still difficult to be widely promoted because of unfamiliar surgical approaches and high infection rate. Hence, the majority of surgeons prefer the posterior approach. However, in fact, the front part of the screws will swing laterally with the posterior compression force of the screw ends, resulting in incomplete reduction of the atlas anterior arch fractures. Atlantoccipital joints and atlantoaxial facet joints are load-bearing synovial joints, which should be the consistent with the treatment of intra-articular fractures of the limbs. Anatomical reduction must be achieved as far as possible to maintain good function [17].

Based on this, Zhang et al. [17] and Rainer et al. [18] firstly proposed and systematically described the principle of posterior osteosynthesis of atlas fractures using a monoaxial lateral mass screw-rod system, which achieved satisfactory anatomical reduction of anterior arch fracture of atlas. However, the widely used posterior cervical screw-rod system has a high internal fixation notch. When using long-tail monoaxial pedicle screw for reduction, the long-arm sleeve needs to be set at a large swing angle on both sides of the screw tail to achieve lever reduction. Then the compression pliers are clamped on the outside of the two sleeves to implement compression reduction and gradually lock the screw plug. In fact, this procedure is difficult to perform. Due to the obstruction of the paravertebral muscles on both sides of the incision, and the large outward swing angle of the long arm sleeve, it is difficult to clamp such a width at both ends of the compression forceps under the condition of the lacked good focus. The U-shaped slot of the monoaxial screw is angled with the transverse connecting rod. If the screw cannot maintain the pressure during locking, the screw will shift to both ends of the connecting rod, and the transverse connecting rod will also be difficult to control rotation. Therefore, this system is not only difficult to operate, but also prone to damage the atlantoaxial sinus, which may lead to massive bleeding. In addition, the length of the connecting rod is also difficult to control, often resulting in insufficient length and rework, or excessive length stimulation of the paravertebral muscles, coupled with the high profile of the screw tail, which is prone to postoperative paravertebral muscle reduction difficulties, chronic bursitis and neck pain. To achieve ideal reduction effect, surgical operation needs to be simplified to improve surgical safety and reduce surgical trauma. On this basis, we designed a posterior low-profile screw-plate system, providing a new therapeutic strategy for the treatment of atlas fractures.

The system is composed of two arc-shaped fixed plates made of titanium alloy connected by adjusting nuts, and a tapered screw hole can be embedded into in the screws at both ends of the side block (Fig. 1). The axial inclination angle of the oblique conical screw hole (15°-20°) is slightly larger than the actual angle of the screw path. The screw is not tightened temporarily after implantation. The relative position of the two plates is first adjusted and locked by adjusting nut connected to the two plates, and the posterior arch fracture of the atlas can be reduced directly under compression reduction. Then bilateral lateral mass screws are tightened. With the conical structures of the screw tail, the relative position will gradually matches the conical hole on the arc-shaped plate. When locking the screws, the anterior part of the screw drives the lateral mass of the atlas forward and inward to achieve compression reduction of the anterior atlas arch fracture. The reduction process is the same principles as that of the monoaxial lateral mass screw-rod system. If the patient has a lateral mass coronal fracture, compression reduction of the lateral atlas mass can be replaced with a half-thread lag screw. Undoubtedly, C1-C2 or C0-C2 fusion is still necessary for patients with irreducible lateral mass fractures, such as sagittal fracture and comminuted fracture of the lateral mass, which will inevitably lead to atlantooccipital or atlantoaxial traumatic osteoarthritis. In addition, due to the certain head tilt angle of C1 screw in the actual screw placement process, collision between the plate and the upper edge of the axial lamina may occur, thus affecting the therapeutic effect. Therefore, during the design process of the plate system, the axis direction of the screw hole and the central plane direction of the plate have a certain angle (10°-15°), so that the plate can be directly attached to the atlas posterior arch after screw placement, thereby avoiding the extrusion of the upper edge of the plate on the atlas posterior arch, and reducing the extrusion of the plate on the axial lamina (Fig. 2 D).

Using this screw-plate system, the surgical operation can be simplified to improve the reduction effect of the atlas anterior arch fracture, so as to minimize the screw profile and reduce the incidence of postoperative symptoms, such as severe occipital nerve stimulation, chronic bursitis and neck pain. In this study, all the 10 patients achieved satisfactory reduction, without postoperative complications related to plate and incision infection, and obvious postoperative symptoms of significant occipital nerve stimulation. At the last follow-up, cervical flexion, extension and rotation were well preserved, and 3D CT showed bone fusion without signs of instability or complications. However, in fact, the curvature of this plate is fixed, which cannot be completely consistent with the curvature of the posterior atlas arch. Therefore, the plate may not fit the posterior atlas arch during the operation, resulting in the compression of the posterior atlas arch, and further displacement of posterior arch fracture, which needs to be gradually optimized in the subsequent process.

As mentioned previously, the primary limitations of this study are the small sample size, the lack of quantitative range of C0-C1 and C1-C2 joints, and the possibility of selection bias. The safety and efficacy of this new technique need to be evaluated in more cases in the future.

## Conclusion

Posterior osteosynthesis with this new screw-plate system can provide a new therapeutic strategy for unstable atlas fractures with simple and almost satisfactory reduction. Multicenter prospective studies of more cases need to be evaluated in the future due to the limited samples of this research.

## Data Availability

The datasets used and analyzed during the current study are available from the corresponding author on reasonable request.

## Abbreviations

TAL: Transverse atlantal ligament
LMD: Lateral mass displacement
AADI: Anterior atlantodens interval
VAS: Visual analogue scale
CT: Computed tomography
MRI: Magnetic resonance imaging
